# Delayed Burr Hole Evacuation Compared With Acute Craniotomy for Acute Subdural Hematoma in Older Patients With Low-Energy Trauma

**DOI:** 10.7759/cureus.63057

**Published:** 2024-06-24

**Authors:** Robert S Graham, Ashwin Ghadiyaram, Noah Feld, Alper Dincer, Dean M Leonard, Erica Johnson, Charles F Opalak, William C Broaddus

**Affiliations:** 1 Department of Neurosurgery, Virginia Commonwealth University Health System, Richmond, USA; 2 Department of Psychiatry and Human Behavior, Thomas Jefferson University, Philadelphia, USA; 3 Department of Neurosurgery, Tufts Medical Center, Boston, USA; 4 Department of Neurosurgery, West Virginia University, Morgantown, USA; 5 Neurosurgery, Prisma Health Southeastern Neurosurgical and Spine Institute, Greenville, USA

**Keywords:** midline shift, hemorrhage, glasgow coma scale, craniotomy, burr hole washout, subdural hematoma

## Abstract

Background

Acute subdural hematomas commonly require emergent surgical decompression by craniotomy. There is currently limited research on alternative surgical strategies in the elderly population. This study investigates delayed surgical intervention for stable patients with low-energy trauma presenting with acute subdural hematomas.

Methodology

In this retrospective chart review, 45 patients over the age of 55 presenting with acute subdural hematomas with a Glasgow Coma Scale score greater than or equal to 13 in the setting of low-energy trauma were selected. Additionally, included patients had a maximal hematoma thickness of >10 mm and/or a midline shift size of >5 mm per the current Brain Trauma Foundations guidelines for surgical intervention of subdural hematomas. The study was performed at a large tertiary care center, with records being examined from 1995 to 2020. Comparison groups were immediate craniotomy (within 24 hours) or delayed burr hole (minimum of 48 hours passing since the initial presentation). Primary outcomes included minor complications, major complications, any complications, and any complications with mortality excluded. There was no significant difference in mortality between the two cohorts.

Results

The immediate craniotomy group consisted of 16 patients, while the delayed burr hole group consisted of 29 patients. The results demonstrated a statistically significant increase in the incidence of any complication including mortality (relative risk (RR) = 3.17, 95% confidence interval (CI) = 1.71-5.88, p < 0.0001), major complications (RR = 2.33, 95% CI = 1.07-5.07, p = 0.031), and minor complications (RR = 2.42, 95% CI = 1.02-5.74, p = 0.041) in the immediate craniotomy group compared to the delayed burr hole group.

Conclusions

Our study demonstrates the decreased risk of major and minor complications for delayed burr hole evacuation in stable patients >55 years old presenting with low-energy trauma and subdural hematoma. The results suggest that for this population of patients, it appears to be beneficial to delay surgery if the patient’s clinical situation allows.

## Introduction

Acute subdural hematomas (aSDHs) are a common and sometimes lethal presentation of traumatic brain injury (TBI) [1]. The incidence of TBI is highest, and increasing, in the elderly population, who are particularly susceptible to TBIs secondary to falls, with 45% of patients >65 years of age with any severity of TBI presenting with computerized tomography (CT) evidence of SDH [2].

In the elderly, even minor trauma may cause sheering forces to the vasculature of the brain, leading to a hematoma between the dura and brain parenchyma. The hematoma can result in global and focal ischemia, coagulopathy, and delayed hematomas [3]. These bleeds are often complicated by co-existing contusional hematomas, diffuse axonal injuries, and subarachnoid hemorrhage. SDHs are associated with significant morbidity and mortality, with an estimated 32% mortality and 33% recurrence rate [4]. Increasing age remains a poor prognostic factor for patients presenting with aSDHs [5].

Evacuation of the hematoma with a craniotomy is often necessary to reduce mortality, mass effect, and secondary ischemic injury. However, surgical intervention is independently associated with significant morbidity and mortality [6-9]. As the population ages, neurosurgeons are often faced with the dilemma of surgical versus expectant management in a patient population with significant medical comorbidities and poor prognostic factors.

Recent studies have shown that delaying surgery for several days could be beneficial in certain circumstances, including patients who are elderly or have a mild TBI on the Glasgow Coma Scale (GCS) score on presentation [10,11]. This delay may be critical to stabilize and optimize patients before surgical intervention and allows for thrombolysis of the clot. However, this research remains sparse and limited due to small sample sizes. Our clinical experience with elderly patients with aSDHs and good neurological function suggested that delayed treatment with burr hole evacuation could produce a good or better outcome than subjecting the patient to an emergent craniotomy for the evacuation of the aSDH.

This study seeks to investigate our hypothesis that older patients presenting with an aSDH in the setting of a low-energy trauma and high GCS score will have decreased complications and mortality when treated with a delayed burr hole evacuation compared to early craniotomy.

## Materials and methods

A retrospective study was performed using patients presenting to a single level I trauma center. The Virginia Commonwealth University (VCU) Health trauma registry was queried for adults who presented with aSDHs at VCU Health from 1995 to 2020. Criteria for inclusion included age greater than 55 years, initial presenting GCS equal to or greater than 13, surgical intervention craniotomy or burr hole, and a maximal subdural thickness of >10 mm or midline shift of >5 mm. Exclusion criteria included craniotomies performed in a delayed fashion (greater than 24 hours after presentation) or burr holes performed earlier than 48 hours from the initial presentation. The sample was divided into the following two groups for comparison: patients receiving an open craniotomy within 24 hours of presentation (early craniotomy group) and patients receiving a burr hole washout at least 48 hours after initial presentation (delayed burr hole group). These time frames were chosen to compare patients taken early for SDH evacuation with those in whom a delayed approach with burr hole drainage was planned.

Data collected for each patient included information from the patient’s initial presentation to the emergency department until the patient’s six-month follow-up in the outpatient clinic after surgery. Demographic data were collected for each patient, including age, gender, race, and smoking status. CT characteristics of the aSDH were collected, including subdural chronicity, associated midline shift, and subdural size. Postoperative complications were collected and separated into major and minor categories. Major complications included subdural reaccumulation, hematomas, repeat surgery, surgical site infection (SSI), sepsis, seizure, intensive care unit (ICU) readmission, intubation, stroke, and mortality. Minor complications included urinary tract infection (UTI), pneumonia, and deep venous thrombosis (DVT).

The primary outcomes measured were minor complications, major complications, any complications, and any complications excluding those resulting in mortality. Categorical variables were described with frequency and percentages. Continuous variables were described with mean and standard deviation and categorical variables between the two groups were compared using the chi-squared analysis. Continuous variables between the groups were compared using either the Student’s t-test for normal distribution or the Wilcoxon signed-rank test for non-normally distributed data. Differences in primary and secondary endpoints were calculated using the chi-squared analysis and relative risk (RR). Multivariate regression analysis was utilized to determine predictors of postoperative outcomes. All statistical analysis was performed using Statistical Analysis Software (SAS) (Cary, NC, USA) and R (Version 4.1.0). P-values refer to two-tailed tests with a significance level of 0.05.

## Results

A total of 45 patients met the inclusion criteria for this study. Patient demographics are summarized in Table 1. Of the 45 patients, 16 underwent emergent craniotomies and 29 underwent burr hole drainage procedures. The early craniotomy group included more males (n = 13, 81%) compared to females (n = 3, 19%), whereas the delayed burr hole group had one less male (n = 14, 48%) than females (n = 15, 52%; p = 0.03). The samples were otherwise similar in age (p = 0.86), race (p = 0.68), and smoking status (p = 0.53). Subdural characteristics are summarized in Table 2. Of the subdural characteristics for the early craniotomy and delayed burr hole group, presenting GCS (14.31 ± 0.87 vs 14.86 ± 0.44, p = 0.007) and size of midline shift (9.8 ± 6.03 vs. 5.37 ± 3.78; p = 0.004) met statistical significance. Other subdural characteristics including chronicity (p = 0.84), hematoma size (17.04 ± 5.94 vs. 17.92 ± 5.88; p = 0.63), and presence of midline shift (75% vs. 83%; p = 0.53) were not statistically different.

**Table 1:** Demographics of patients included in the study and separated by group. * denotes statistical significance (p < 0.05). GCS = Glasgow coma scale; COPD = chronic obstructive pulmonary disease

Demographic	Treatment group		
	Early craniotomy (n, %)	Delayed burr hole (n, %)	P-value
n	16 (36%)	29 (64%)	
Age	73 ± 10.21	73 ± 10.44	0.86
Gender			
Male	13 (81%)	14 (48%)	0.03*
Female	3 (19%)	15 (52%)	
Race			
White	11 (69%)	16 (56%)	0.68
African American	4 (25%)	9 (31%)	
Asian	0 (0%)	2 (7%)	
Other	1 (6%)	1 (3%)	
Unknown	0 (0%)	1 (3%)	
Presenting GCS	14.31 ± 0.87	14.86 ± 0.44	0.007*
Smoking status			0.53
Current	4 (25%)	5 (17%)	
Non-smoker	9 (56%)	21 (73%)	
Unknown	3 (19%)	3 (10%)	
Comorbidities			
Diabetes mellitus	5 (31%)	6 (21%)	0.43
Hypertension	13 (81%)	25 (86%)	0.66
Hyperlipidemia	12 (75%)	12 (41%)	0.03*
Myocardial Infarction	5 (31%)	3 (10%)	0.079
Heart failure	1 (6%)	3 (10%)	0.64
COPD	0 (0%)	0 (0%)	
Antiplatelets	7 (44%)	13 (45%)	0.94
Anticoagulation	6 (38%)	5 (17%)	0.13
Disposition			0.11
Home	3 (19%)	14 (48%)	
Dead	1 (6%)	2 (7%)	
Palliative	2 (13%)	0 (0%)	
Skilled nursing facility	7 (43%)	9 (31%)	
Inpatient physical therapy	3 (19%)	2 (7%)	
Other	0 (0%)	2 (7%)	

**Table 2:** Subdural characteristics based on initial presenting computerized tomography (CT) head scan. * denotes statistical significance (p < 0.05).

Subdural characteristics	Treatment group		
	Early craniotomy (n, %)	Delayed burr hole (n, %)	P-value
Chronicity			0.84
Acute	9 (56%)	15 (52%)	
Acute on chronic	6 (38%)	13 (45%)	
Chronic	0 (0%)	0 (0%)	
Unknown	1 (6%)	1 (3%)	
Hematoma size (mm)	17.04 ± 5.94	17.92 ± 5.88	0.63
Midline shift	12 (75%)	24 (83%)	0.53
Midline shift size (mm)	9.82 ± 6.03	5.37 ± 3.78	0.004*

**Figure 1:**
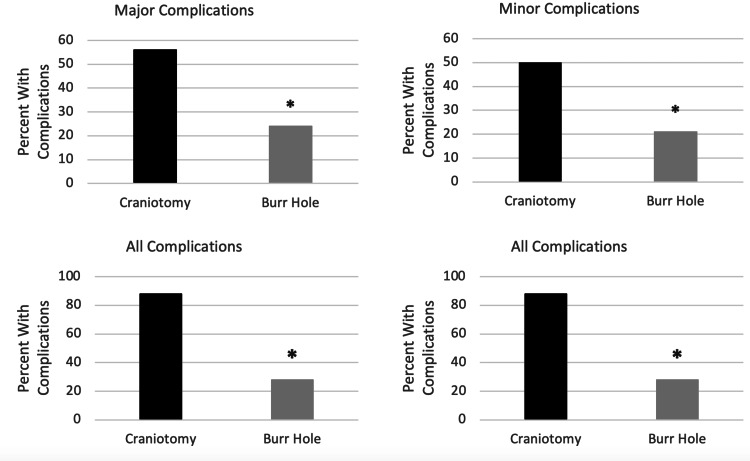
Major, minor, and any complications (with/without mortality) for patients receiving emergent craniotomy or delayed burr hole treatment for a subdural hematoma. * denotes statistical significance (P < 0.05)

Postoperative complications for the early craniotomy and delayed burr hole cohorts are statistically summarized in Table 3 and graphically summarized in Figure 1. Overall, 14 of the 16 (88%) cases of early craniotomy experienced a complication compared to eight of the 29 (28%) delayed burr hole patients experiencing a complication (RR = 3.17 [1.71-5.88]; p = 0.0001). Nine of the 16 (56%) early craniotomies experienced a major complication while seven of the 29 (24%) patients experienced a major complication in the delayed burr hole group (RR = 2.33 [1.07-5.07]; p < 0.031) (Figure 1). In the early craniotomy group, eight of the 16 (50%) patients experienced a minor complication and six of the 29 (21%) in the delayed burr hole group experienced a minor complication (RR = 2.42 [1.02-5.74]; p = 0.041) (Figure 1). Of the individual complications, DVT had a statistically significant difference among the groups (p < 0.016). Three of the 16 craniotomy patients experienced a DVT during their hospital course compared to none of the 29 patients in the delayed burr hole group. Reintubation during hospital stay trended toward statistical significance (p < 0.051). Two of the 16 early craniotomy patients had to be reintubated during their hospital stay, while none of the 29 patients had to be reintubated in the delayed burr hole group. In the early craniotomy group, three of the 16 patients had to undergo a repeat washout. In comparison, two of the 29 patients had to undergo a repeat washout in the delayed burr hole group (p < 0.23). For mortality, in the early craniotomy group, three of the 16 patients (19%) died. In comparison, the delayed burr hole group had two out of the 29 patients who died (7%; p = 0.22). There was no statistically significant difference between the cohorts in terms of ICU length of stay.

**Table 3:** Frequency of complications and ICU length of stay of early craniotomy and delayed burr hole groups. Relative risk calculated for major complications, minor complications, and all complications with/without mortality. * denotes statistical significance (p < 0.05). ICU = intensive care unit; UTI = urinary tract infection; DVT = deep venous thrombosis; RR = relative risk; CI = confidence interval

Postoperative complications	Treatment group			
	Early craniotomy (n, %)	Delayed burr hole (n, %)	RR (95% CI)	P-value
Major	9 (56%)	7 (24%)	2.33 (1.07–5.07)	0.031*
Subdural reaccumulation	3 (19%)	4 (14%)		0.66
Hematomas	0 (0%)	0 (0%)		N/A
Repeat surgery	3 (19%)	2 (7%)		0.23
Surgical site infection	1 (6%)	0 (0%)		0.17
Sepsis	0 (0%)	1 (3%)		0.45
Seizure	3 (19%)	1 (3%)		0.08
ICU readmission	1 (6%)	3 (10%)		0.64
Intubation	2 (13%)	0 (0%)		0.051
Stroke	0 (0%)	0 (0%)		N/A
Mortality	3 (19%)	2 (7%)	2.72 (0.51–14.41)	0.22
Minor	8 (50%)	6 (21%)	2.42 (1.02–5.74)	0.041*
UTI	5 (31%)	7 (24%)		0.61
Pneumonia	0 (0%)	2 (7%)		0.28
DVT	3 (19%)	0 (0%)		0.016*
All	14 (88%)	8 (28%)	3.17 (1.71–5.88)	0.0001*
All (no mortality)	13 (81%)	7 (24%)	3.37 (1.69–6.69)	0.0002*
ICU length of stay (days)	7.69 ± 9.67	5.00 ± 5.97		0.33

Multivariate regression analysis unveiled significant predictors of postoperative complications in the cohort who underwent delayed burr hole treatment. Notably, a history of diabetes mellitus (p = 0.016) and hypertension (p = 0.029) emerged as strong indicators (Table 4). Moreover, the degree of midline shift resulting from SDH (p = 0.0498) stood out as a robust predictor of postoperative subdural reaccumulation (Table 5). Additionally, factors such as the initial GCS measurement upon emergency department admission (p = 0.013) and a history of smoking (p = 0.022) strongly correlated with ICU length of stay in these patients (Table 6).

**Table 4:** Multivariate regression for occurrence of any postoperative complications in patients who underwent delayed burr hole treatment. * denotes statistical significance (p < 0.05). SDH = subdural hematoma; GCS = Glasgow Coma Scale

Variable	Coefficient	Standard error	t statistic	P-value
Intercept	4.22	3.33	1.27	0.24
Age	0.016	0.016	1.014	0.34
Sex	0.37	0.24	1.54	0.16
Smoking	-0.0056	0.0077	-0.73	0.49
Race	0.0099	0.13	0.076	0.94
Cause of SDH	0.11	0.075	1.48	0.18
GCS in the emergency department	-0.49	0.29	-1.69	0.13
Degree of midline shift	-0.047	0.21	-0.23	0.83
Hematoma chronicity	0.16	0.31	0.53	0.61
Hematoma size	-0.057	0.026	-2.17	0.062
Hematoma bilaterality	-0.094	0.18	-0.53	0.61
Preoperative GCS	0.20	0.17	1.17	0.28
History of diabetes mellitus	0.83	0.28	3.031	0.016*
History of hypertension	-0.85	0.32	-2.65	0.029*
History of hyperlipidemia	-0.14	0.22	-0.63	0.55
History of myocardial infarction	-0.65	0.65	-0.996	0.35

**Table 5:** Multivariate regression for subdural reaccumulation in patients who underwent delayed burr hole treatment. * denotes statistical significance (p < 0.05). SDH = subdural hematoma; GCS = Glasgow Coma Scale

Variable	Coefficient	Standard error	t statistic	P-value
Intercept	3.014	2.47	1.22	0.26
Age	0.015	0.012	1.28	0.24
Sex	-0.083	0.18	-0.47	0.65
Smoking	-0.0085	0.0057	-1.50	0.17
Race	0.12	0.096	1.29	0.23
Cause of SDH	-0.010	0.055	-0.18	0.86
GCS in the emergency department	-0.35	0.22	-1.64	0.14
Degree of midline shift	-0.35	0.15	-2.31	0.0498*
Hematoma chronicity	0.12	0.23	0.52	0.62
Hematoma size	-0.029	0.019	-1.50	0.17
Hematoma bilaterality	0.061	0.13	0.46	0.66
Preoperative GCS	0.16	0.13	1.25	0.25
History of diabetes mellitus	0.25	0.20	1.24	0.25
History of hypertension	-0.49	0.24	-2.074	0.072
History of hyperlipidemia	-0.17	0.16	-1.044	0.33
History of myocardial infarction	-0.21	0.48	-0.44	0.67

**Table 6:** Multivariate regression for ICU length of stay in patients who underwent delayed burr hole treatment. * denotes statistical significance (p < 0.05). SDH = subdural hematoma; GCS = Glasgow Coma Scale

Variable	Coefficient	Standard error	t statistic	P-value
Intercept	139.01	28.72	4.84	0.0029*
Age	-0.066	0.17	-0.39	0.71
Sex	2.45	2.031	1.21	0.27
Smoking	7.61	2.47	3.082	0.022*
Race	-0.65	1.21	-0.54	0.61
Cause of SDH	0.068	0.65	0.10	0.92
GCS in the emergency department	-8.99	2.59	-3.47	0.013*
Degree of midline shift	-1.074	1.98	-0.54	0.61
Hematoma chronicity	-2.016	2.76	-0.73	0.49
Hematoma size	-0.26	0.23	-1.15	0.30
Hematoma bilaterality	1.13	1.70	0.67	0.53
Preoperative GCS	0.97	1.45	0.67	0.53
History of diabetes mellitus	3.036	2.37	1.28	0.25
History of hypertension	-5.82	3.38	-1.72	0.14
History of hyperlipidemia	-1.25	1.91	-0.65	0.54
History of myocardial infarction	-3.44	5.73	-0.60	0.57

## Discussion

aSDHs are a common neurosurgical emergency with an increasing presence in the elderly population. Early surgical evacuation of the SDH is associated with significant morbidity and mortality, especially within the elderly population [4]. This study utilized the VCU Trauma database to perform a retrospective analysis to compare immediate craniotomy versus delayed burr hole evacuation in elderly patients presenting with a subdural in the setting of a low-energy trauma and high GCS score.

The two groups were largely similar in demographics, comorbidities, and subdural characteristics. Of note, the early craniotomy group had a ~4 mm greater average midline shift compared to the delayed burr hole group, which was statistically significant. Additionally, we found a statistically significant difference in presenting GCS (14.31 ± 0.87 vs. 14.86 ± 0.44, p = 0.007). The lower average GCS of the craniotomy group could also be related to why these patients underwent an early craniotomy. Among comorbidities listed in Table 1, hyperlipidemia was found to be statistically significant in difference (75% vs. 41%, p = 0.03). While it is unlikely that this played a role in the outcome of the study, it is worth noting.

We uncovered a notable decrease in complications with delayed intervention, marking a significant departure from the outcomes associated with early craniotomy. The group treated with burr hole evacuation demonstrated significantly fewer major, minor, and overall complications compared to their counterparts. Remarkably, our analysis highlighted the pivotal role of the degree of midline shift of the SDH in predicting postoperative subdural reaccumulation, the most prevalent major complication, among patients undergoing delayed burr hole treatment (p = 0.0498). Additionally, within this cohort, the initial GCS measurement upon emergency department presentation exhibited a strong correlation with ICU length of stay (p = 0.013). These findings underscore the value of comprehensive and timely assessment through both physical examination and imaging modalities for optimal preoperative management of this patient population.

The all-complications group included and excluded mortality to further evaluate the differences between the two groups. The results illustrate that whether included or not, the delayed burr hole group had fewer instances of complications. The RR additionally demonstrates that delayed burr hole evacuation is protective compared to early craniotomy. While not statistically significant, it is worth noting that the mortality in the early craniotomy group had three of the 16 patients who expired (19%) and the burr hole group had two of the 29 patients who expired (7%). It should also be noted that among patients with SDH not included in either of these groups, none of the patients who died had been planned for delayed burr hole drainage.

While it was difficult to obtain long-term follow-up data due to differences in documentation and incomplete patient charts (especially those from the early 2000s and prior), we did find a trend toward significance in the difference in patient disposition (p < 0.11). Most specifically, three of the 16 craniotomy patients (19%) and 14 of the 29 burr hole patients (48%) were discharged home (p < 0.051). While not statistically significant, with the addition of more patients in such a study, the difference could prove to be significant.

Previous studies have demonstrated the benefit of delayed burr hole drainage for the elderly population. Akibik et al. demonstrated in their retrospective analysis that delayed management in elderly patients (median age = 77) with high presenting GCS (68% had a GCS of at least 13) benefited from delayed surgery (median = 11 days) [12]. In a study by Watson et al., treatment of chronic SDHs in the elderly population with burr hole drainage resulted in a decreased cost of stay and decreased reoperation rates [13]. While these studies illustrate the benefit of delayed surgical intervention, few studies have compared the efficacy of delayed burr hole drainage in the setting of aSDHs.

The results from this study are consistent with previous findings that elderly patients may benefit from the delayed surgical intervention in the setting of aSDHs. However, the risk versus benefit must be weighed in every clinical situation. The elderly population is particularly prone to morbidity, mortality, and postoperative complications. Recent research has shed light on the risk factors associated with the transformation of traumatic acute subdural hematomas (TASDHs) to chronic subdural hematomas (CSDHs) in elderly patients. For instance, a retrospective analysis by Wasfie et al. identified diabetes, race, and initial disposition as significant risk factors for the subsequent development of CSDH in elderly patients following TASDH [14]. Furthermore, the risks associated with antithrombotic therapy in this patient population should be considered. A meta-analysis by Hart et al. confirmed that utilization of aspirin increases the RR of SDH occurrence in this patient population [15]. These findings underscore the importance of considering not only immediate surgical intervention but also long-term follow-up and risk assessment in this patient population. In a subset of this population, we found that patients over the age of 55 years with a GCS >13 after a low-energy trauma may benefit from a delayed surgical evacuation of the hematoma.

This study has several limitations. The study was conducted as a retrospective analysis at a single-center institution, increasing the risk of selection bias and generalizability. Measurement of other important outcomes, such as quality of life, disability, and long-term mortality, were not included in this study primarily due to limitations in the VCU Trauma database and patient records. Midline shift size was found to be statistically significantly different on the two-sample t-test examination. This is likely secondary to a few outliers among the craniotomy group, as evidenced by the large standard deviation. An additional limitation is the statistically significant difference between GCS and hyperlipidemia among both groups. For GCS, this can likely be explained due to on average craniotomy patients having lower GCS scores, thus potentially prompting immediate surgical intervention. For hyperlipidemia, it likely did not have an impact on the study results itself, and is likely related to the small sample size. One of our limitations when describing the hematoma characteristics was the fact that some of the patient charts before 2010 had incomplete or absent descriptions of the hematoma. As such, the analysis pool was smaller.

## Conclusions

Our study demonstrates that patients over 55 years old with mild TBI and low-energy trauma who were deemed appropriate for delayed management of aSDHs had a more favorable outcome compared to those who underwent immediate surgical intervention. While this may reflect a selection bias for planning delayed management in a more favorable group of patients, it is also possible that by allowing time for stabilization and optimization, healthcare providers can potentially mitigate the risk of postoperative complications in this vulnerable demographic. It is essential to approach each clinical scenario with careful consideration of the individual patient’s characteristics and clinical status, weighing the benefits of delayed intervention against the potential risks. Further research with larger sample sizes in the form of a randomized clinical trial with long-term follow-up data would be required to validate these findings and refine clinical guidelines for managing aSDHs in the elderly population.
